# Female finches prefer courtship signals indicating male vigor and neuromuscular ability

**DOI:** 10.1371/journal.pone.0226580

**Published:** 2020-01-10

**Authors:** Jeffery L. Dunning, Santosh Pant, Karagh Murphy, Jonathan F. Prather

**Affiliations:** 1 Department of Pharmacology, Vanderbilt University, Nashville, Tennessee, United States of America; 2 Department of Zoology & Physiology, Neuroscience Program, University of Wyoming, Laramie, Wyoming, United States of America; Texas Christian University, UNITED STATES

## Abstract

Female songbirds use male song to discriminate among individuals and evaluate their quality as potential mates. Previous behavioral experiments in many species, including the species studied here, have shown that females will solicit copulation in response to song even if no male is present. Those data demonstrate that female mate choice is closely tied to song features, but they leave open the question of which song parameters are most influential in female mate selection. We sought to identify features of male song that are salient for mate choice in female Bengalese finches. Using a novel experimental approach, we simultaneously tested the possible influence of specific notes or note transitions, the number of different note types in the male’s repertoire, the complexity of note content and note sequence, and the stereotypy of note content and note sequence. In additional experiments, we also tested the influence of the pitch and tempo of note production. Our results demonstrate that females generally preferred songs containing increased tempo in the context of species-typical frequency bandwidth, consistent with the idea that females prefer songs that are especially challenging to produce. Female preference for song features that pose a neuromuscular challenge has also been reported in other species. Our data extend those observations into a species that thrives in a laboratory setting and is commonly used in studies of the neural basis of behavior. These results provide an excellent new model system in which to study female preference and the neural mechanisms that underlie signal evaluation and mate choice.

## Introduction

Songs are complex learned vocalizations that male songbirds use as a form of behavioral ornamentation to advertise their quality to potential mates [1]. In many species, including the Bengalese finches (BFs, *Lonchura striata*) we study here, males sing but females cannot sing. Despite their absence of song performance, females are nonetheless as good as or better than males at discriminating fine details of auditory stimuli and evaluating the quality of songs performed by male suitors [2]. Song plays such an important role in female mate preference that females may express copulation solicitation displays (CSDs) in response to songs played through a speaker, even if no male is physically present [3, 4]. Therefore, if we are to understand the set of factors that influence female mate choice, it is essential to define the relation between mate preference and the finely detailed structure of songs that females evaluate to make that decision.

Studies of a variety of species have revealed several song parameters that are especially salient in affecting female mate preference. For example, females commonly prefer songs with longer duration compared to those with shorter duration [5–9]. Those results suggest that females may be evaluating males based on a male’s ability or willingness to sustain a challenging vocalization for a prolonged time. Although studies have implicated duration alone as a salient factor in female preference, longer songs may also contain a greater variety of content. In support of the idea that diversity of song content plays a role in female song preference, females of many species prefer males that sing a greater variety of different vocalizations [6, 7, 10–13]. These data suggest that females may be evaluating males based on the variety of different song behaviors that the male can perform. Female preference has also been associated with the presence of song elements that are especially challenging to produce, such as the “sexy syllables” present in the songs that female canaries find attractive [14, 15] and the specific elements that characterize attractive water pipit songs [16]. Preference for challenging song elements is also evident in the preference of female Lincoln’s sparrows for songs with very rapid trills [17] and the preference of female swamp sparrows for songs containing a combination of broad frequency bandwidth and rapid tempo [18, 19]. Additional studies have also suggested that other features such as stereotypy of song structure [20, 21] and complexity of note sequence [22, 23] may influence female preference.

Previous studies of the songs that females find attractive make it clear that female songbirds use a wide variety of song parameters to guide their mate choice, but it remains challenging to identify the contribution of any single parameter to the decision of mate choice in any given species. Our previous work revealed a means of measuring the mate preferences expressed by individual female BFs [3]. When female BFs are presented a set of songs performed by different males, each female expresses an individual-specific preference for one song more than others [3]. Individual-specific preferences of BF females are stable across time and tests, indicating that preference is related to some aspect of the song itself. It is reasonable to presume that some characteristic of the female BF’s most-preferred song distinguishes it from other less-preferred songs, yet tests have yielded inconclusive results about the relation between female BF preference and the magnitude of specific song properties [22, 24]. Therefore, it remains unknown what role any specific parameter plays in affecting female BF mate choice.

Here we introduce a novel method capable of simultaneously testing the role of many different song parameters in affecting female evaluation of song quality. This approach relies on our previously established behavioral method of identifying each female BF’s most-preferred song from among a set of many conspecific songs [3]. The number of calls performed by each female in response to playback of songs performed by different males is directly related to the number of CSDs performed in response to the same stimuli [3]. Thus, the number of calls in response to each song provides a measure of each female’s mate preference. In the current study, we use the methods of Dunning et al [3] to identify each female’s most-preferred song, then we repeatedly bisect that song and use the first and second halves to construct new artificial stimuli to test the female’s preference for the content of each half. By defining the impact of each song division on the female’s expression of behavioral indicators of mate choice, we tested the hypothesis that female BF mate choice is closely related to the presence, absence or degree of specific song parameters. This novel method affords the ability to test hypotheses that emerge from three models of the features of male song that affect female BF evaluation of song quality: 1) females are responsive to specific song elements or stereotypy of note content, 2) females are responsive to complexity or stereotypy of note sequence, and 3) females are responsive to some global song feature(s) that is broadly conserved throughout a song performance. Insights gained from tests of these models are an important step forward in developing a more complete understanding of how female birds evaluate the subjective value of sensory input and use that information to select a specific mate from among many possible outcomes.

## Materials and methods

### Care and handling of experimental subjects

We performed all experiments using adult (age > 120 days post-hatch) male (n = 11) and female (n = 12) BFs obtained from a commercial breeder or from our breeding colony. We identified males based on the presence of singing behavior. If a bird did not sing during continuous recording for four or more consecutive days, we considered it female. No bird identified as female in this way produced a song at any other point during the experiment. All identifications were confirmed histologically at the end of the experiment, with males identified by the presence of large and obvious sexually dimorphic song-related brain sites [reviewed in 25]. We housed birds in our aviary colony prior to experimentation (15:9 light: dark photoperiod). Before beginning behavioral tests, we removed female subjects from the colony and placed them in sound-attenuating chambers in all-female groups of no more than 8 birds for a minimum of 3 days to isolate those birds and prevent them from hearing song or interacting with male birds [3]. During behavioral testing, we housed birds individually in a wire mesh cage (41 cm x 31 cm x 24 cm) inside a sound attenuation chamber (Industrial Acoustics, model MAC-1). We maintained the 15:9 photoperiod and provided seed and water *ad libitum* during all phases of the study.

We monitored birds daily and minimized the number of times that they were handled, the number of times that they were moved between cages, and the duration of handling in each case. At the conclusion of this research, subjects were housed for use in additional research. All procedures were approved by the University of Wyoming Animal Care and Use Committee (protocol number 20140506JP00106-02), consistent with the Guidelines for the Use of Animals in Research and in compliance with all state and federal regulations governing the housing and use of songbirds.

### Song stimuli and identification of each female’s most-preferred song

A previous publication from our group contains a detailed description of the methods we used to create and present song stimuli, quantify female responses to song stimuli, and quantify each female’s preference for song of an individual male according to the number of calls she produces during playback of song stimuli [3]. Briefly, we recorded the female-directed songs of 11 different males and concatenated the songs of each male into an aggregate song stimulus containing five song performances from that male. The males used to collect those songs were unfamiliar to the females tested here. In behavioral tests of females, birds were housed individually in cages within sound-attenuating chambers (Dunning et al. 2014). We recorded calls using a microphone (Shure model SM57) and confirmed them visually using a camera (General Electric model 45321) inside the cage by associating beak opening with calls during live observations.

We previously established that the number of calls that a female BF produces in response to male song is closely related to her mate preference as measured by the number of CSDs that she produces in response to the same song [3]. Calls have the additional advantage of being more common than CSDs, providing greater resolution in comparing a female’s preference for one song versus another. Therefore, we quantified female preference for male song by counting the number of calls the female produced during presentation of male song through a speaker placed inside the sound-attenuating chamber (Sound Acoustics). In a randomized sequence, we presented songs from each male and identified the female’s “most-preferred song” as the song stimulus that evoked the greatest number of calls.

### Bisecting each female’s most-preferred song and testing preference for song subunits

**Fig 1:**
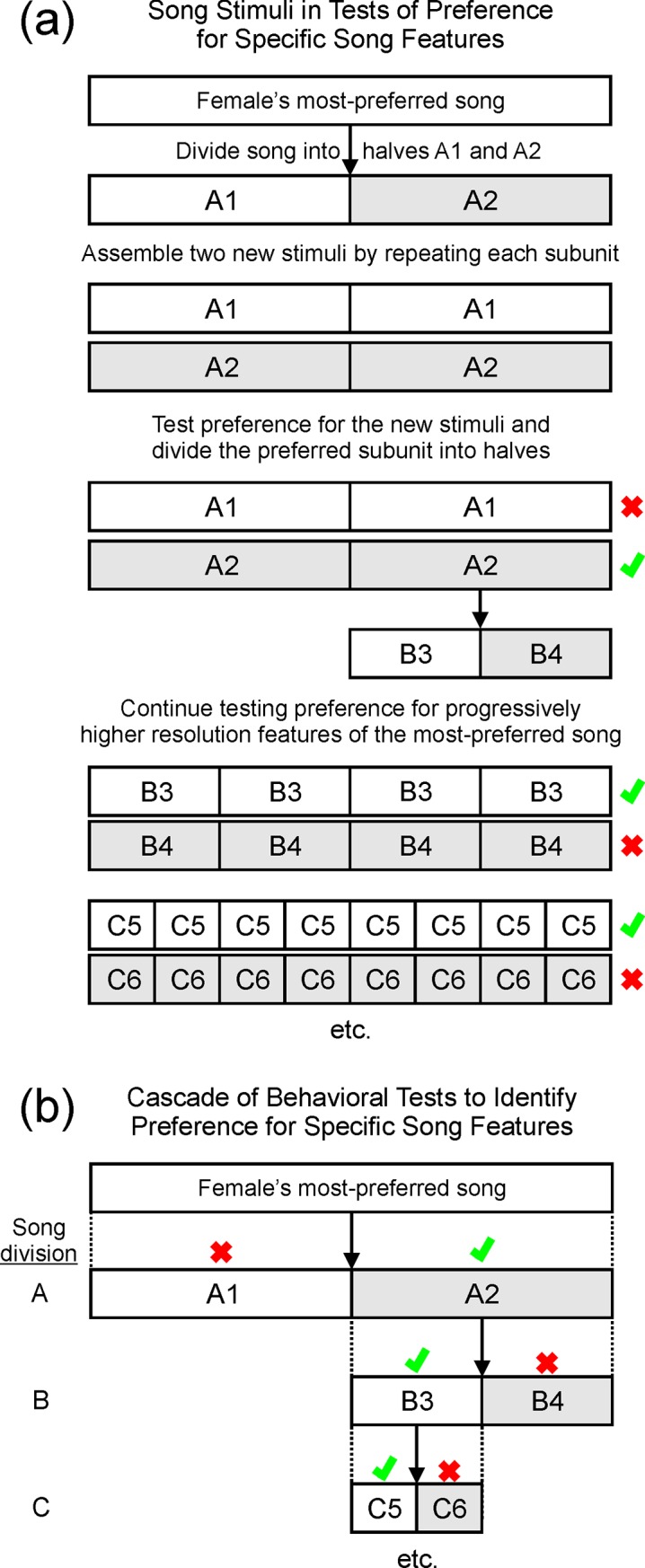
Testing female preference for features present in subunits of the natural song. **(a)** We bisected each female’s most-preferred song into two subunits (A1 and A2 in this example). We repeated each subunit to form two new stimuli with the same duration as the original song (A1-A1 and A2-A2). We tested the female’s response to each of those new stimuli and noted which she preferred (indicated by a check mark). With the preferred subunit identified (A2 in this example), we bisected it again to form two new subunits (B3 and B4) and tested preference for those stimuli in the same way as in the previous stimuli. With the preferred subunit identified (B3, indicated in this and subsequent cases by a check mark), we bisected it again (C5 and C6), formed new stimuli, and tested preference using those stimuli. **(b)** We repeated that process until the bird stopped responding to stimuli or until the song was subdivided down to the level of an individual note.

We based our approach on the idea that if expression of behavioral indicators of mate choice (e.g., calls) were closely linked to the presence of some superlative song element, such as an especially attractive note or note transition, then doubling the density of that song element should double the degree to which the female expressed those behaviors. Similarly, removing that superlative element should result in cessation of those behaviors. To implement that approach, we bisected each female’s most-preferred song and used those two halves (A1 and A2 in Fig 1A) to assemble two new stimuli (songs were bisected so that no individual note was split; the song was bisected within the internote interval closest to the exact midpoint). To preserve song duration, we repeated each subunit to create stimuli with the same duration and tempo as the natural song. Intervals introduced between stimulus subunits were assigned a duration equal to the mean internote interval of the natural song. We then tested the female’s preference for those two new stimuli (A1-A1 vs. A2-A2 in Fig 1A). For the stimulus that evoked the greater response (A2-A2 in Fig 1A, as indicated by a green check in examples throughout Fig 1A and 1B), we bisected the corresponding portion of the original song into subunits that were one quarter of the duration of the original most-preferred song (B3 and B4 in Fig 1A). We repeated those song-quarters four times (B3-B3-B3-B3 and B4-B4-B4-B4) so that they formed two new stimuli of equal duration and tempo as the original song and the duration of the stimuli created by the previous bisection. We used those stimuli in behavioral tests of song preference, and the component that evoked the greater response (B3 in Fig 1A) was again bisected (into C5 and C6 in Fig 1A) and used to make another set of two new song stimuli. We repeated that process of testing, bisecting and retesting (Fig 1B) until females no longer responded to one or both of the new song stimuli (i.e., no calls in response to the stimulus), or until the song was no longer able to be divided (i.e., that the relevant subunit of the original song was down to the level of a single note), or until the bird ceased participating in the behavior test (criteria to establish participation are elaborated below).

To assess whether the bird was participating in the behavioral test, we measured the female’s response to the original, undivided version of her most-preferred song 5 minutes prior to the start of behavioral testing. In order to proceed, we required that the female must be responsive to the natural, undivided song (criteria to define participation in the behavioral test were taken from Dunning et al. 2014: females called at least 10 times in response to the most-preferred song or at least 4 times each to the most-preferred song and the song of at least one other male). Those data served as a positive control that the bird was in a state where she was capable of reporting her song preference. At the termination of the experiment (i.e., if the bird ceased responding to song stimuli), we again tested the female’s response to the natural version of her most-preferred song. That test enabled us to disambiguate cessation of response due to the female simply ceasing to respond at all versus cessation of response due to elimination of some attractive feature. We never encountered a situation in which a female was unresponsive to the natural song but responsive to the bisected song stimuli. Preference testing ended when a female completed five successful trials in response to bisected song stimuli.

### Quantifying behavioral responses across levels of song bisection

To compare behavioral results across levels of song division, we created a metric of the strength of response at each level of song bisection. We counted the number of calls that the female produced in response to the more-preferred stimulus at each level of song division (e.g., responses to A2, B3 and C5 in Fig 1B). We then expressed the magnitude of response at each level of song division as a percentage of the total of all responses to all of the preferred stimuli across all tests in which the bird was tested. This allowed us to compare the strength of response at each level of song division (labeled song division N to permit comparison across birds) versus the strength of response at the immediately subsequent level of song division (labeled song division N+1).

### Modeling possible relations between song parameters and song preference

To determine whether preference(s) for one or more specific song features were evident in our data, we modeled the expected results for each of several possible preferences (e.g., preference for specific song features, preference for stereotypy, etc.) and compared our observed results to the outcomes of those models. *Specific song element*: If female preference were closely related to the presence of a specific element in the natural song (e.g., a “sexy syllable” or a specific set of notes or note transitions), then the density of that element should have become twice as great with each new concatenated stimulus resulting from bisection. That would have resulted in a doubling of the response strength between each song division N and each subsequent division N+1. Such a scenario would be evident as a linear relation with slope of 2 (Model 1). *Stereotypy of note content or note sequence*: If we imagine a song that has note sequence ABCDEFGH, then the bisected and reassembled stimuli would have sequences ABCDABCD and EFGHEFGH. In that and each subsequent bisection and reassembly, the stereotypy at song division N+1 will be double that at song division N. Therefore, strong preference of females for stereotypy of male song would also be evident as a linear relation with slope of 2 (Model 1). *Complexity of note content or note sequence*: Bisection of the natural song and doubling of the song halves to create new song stimuli results in a reduction of song complexity. If female preference were closely related to song complexity, each song bisection would decrease the attractiveness of the stimulus. This scenario would be also evident as a linear relation with slope of ½ (Model 2). *Repertoire of note types*: The number of different note types present in the song stimulus is halved with each bisection and reassembly. Strong preference of females for a large repertoire of note types would also be evident as a linear relation with slope of ½ (Model 2). *Global song feature*: If the salient song feature were present throughout the natural song (e.g., pitch or tempo), then it should be present in roughly equal amounts in the natural song as in each of the bisected and reassembled stimuli. Such a scenario would be evident as a linear arrangement with slope of 1 (Model 3). Such a result would indicate that some broadly conserved feature of song was related to the female’s response to that song, but additional testing would be necessary to test hypotheses about the role of any individual feature.

### Testing the role of song frequency and tempo in female preference

**Fig 2:**
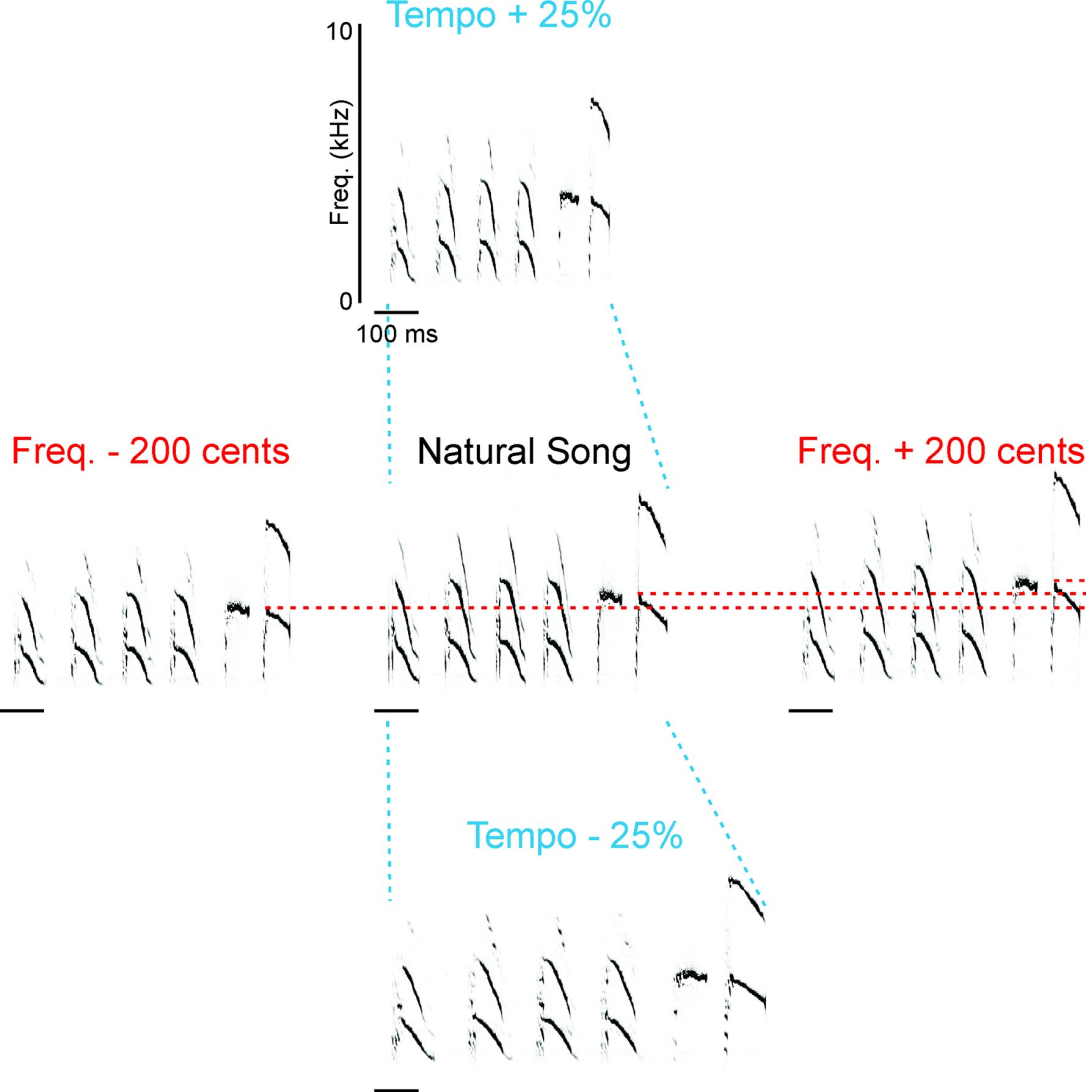
We modified the frequency or the tempo of song stimuli independently. Using the natural performance of each female’s most-preferred song (“natural song”, center panel) as the basis for each set of stimuli, we manipulated either frequency (red font, panels arranged in a row) or tempo of the song recording (blue font, panels arranged in a column). Dashed blue lines highlight the differences in duration of stimuli with altered tempo. Dashed red lines highlight the differences in frequency of the last note in each song. (scale bars = 100 ms and y-axis scales are the same in all panels).

To investigate how female mate choice may be influenced by song features that are present throughout the song stimulus, we used commercial software (ChronoTron Pro, Ianier Munoz) to manipulate independently the pitch or the tempo of each female’s most-preferred song (Fig 2). We manipulated pitch in increments of -200, -100, -50, +50, +100 and +200 cents, and we manipulated tempo to -50, -25, +10, +25, +50 and +100 percent of the natural tempo of each song (Table 1). Thus we tested the role of changes in each parameter within and outside of the range of values found in natural BF songs. Because changing tempo affected not only the tempo itself but also the rate of frequency modulation (FM) in individual notes, we created another set of stimuli to investigate the possible influence of FM. We took the notes that were shortened by increased tempo and separated them by intervals of silence such that the notes composed a song with the same tempo as the original, natural song (custom software in Matlab). This stimulus thus contained the same FM as the accelerated version of the song and the same tempo as the natural version of the song, enabling us to control for changes in FM in our investigation of the role of tempo in affecting female song preference.

**Table 1:** Experimental changes in properties of the natural song type.

	Experimental Manipulation							
Parameter	Tempo—50%	Tempo—25%	Natural Song	Tempo + 10%	Tempo + 25%	Tempo + 50%	Tempo + 100%	p-value
**Tempo**	**0.50 + 0.01**	**0.75 + 0.01**	**1.00 + 0.00**	**1.11 + 0.01**	**1.26 + 0.01**	**1.51 + 0.01**	**2.01 + 0.01**	**< 0.001**
Mean Freq	1.01 + 0.02	1.00 + 0.01	1.00 + 0.00	1.04 + 0.02	1.02 + 0.01	0.98 + 0.02	0.98 + 0.01	0.18
Max Freq	1.02 + 0.02	1.01 + 0.01	1.00 + 0.00	1.01 + 0.01	1.01 + 0.01	1.01 + 0.01	1.00 + 0.01	0.98
Min Freq	0.98 + 0.07	1.03 + 0.07	1.00 + 0.00	0.99 + 0.04	1.14 + 0.09	0.90 + 0.03	0.92 + 0.11	0.26
Parameter	Freq—200	Freq—100	Freq—50	Natural Song	Freq + 50	Freq + 100	Freq + 200	p-value
Tempo	1.00 + 0.01	1.01 + 0.01	1.01 + 0.01	1.00 + 0.00	1.01 + 0.01	1.01 + 0.01	1.01 + 0.01	0.67
**Mean Freq**	**0.88 + 0.02**	**0.97 + 0.01**	**1.02 + 0.01**	**1.00 + 0.00**	**1.03 + 0.04**	**1.09 + 0.01**	**1.13 + 0.02**	**< 0.001**
**Max Freq**	**0.95 + 0.03**	**0.96 + 0.01**	**0.98 + 0.01**	**1.00 + 0.00**	**1.03 + 0.02**	**1.07 + 0.01**	**1.12 + 0.02**	**< 0.001**
Min Freq	1.06 + 0.08	1.05 + 0.09	1.08 + 0.09	1.00 + 0.00	1.01 + 0.08	1.14 + 0.11	1.03 + 0.07	0.91

All values are expressed as percent of the corresponding value in the natural song stimulus (mean ± SE; n = 6 songs). Values in bold font indicate cases of significant change in the measured parameter (p < 0.05 in a one-way ANOVA; Bonferroni correction is not necessary because measurements were taken from different songs). Experimental manipulations of frequency are reported in cents.

### Statistical analysis

**Fig 3:**
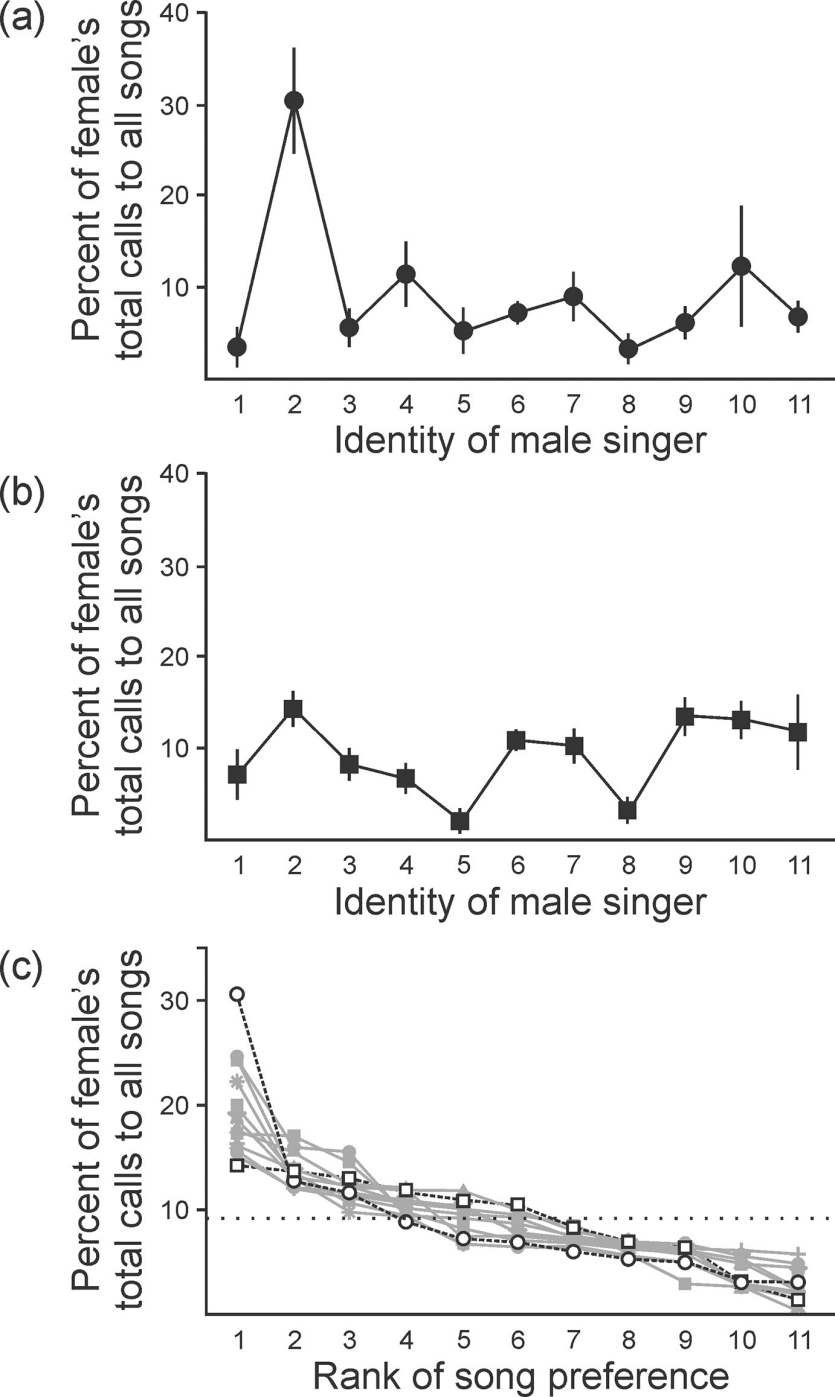
Females difference in their song preferences, but individuals were consistent in their preferences across time and tests. **(a)** Consistent with our previous study of female BFs mate choice (Dunning et al. 2014), some females were very selective in their preference for the song of an individual males (n = 1 bird; points indicate mean of 5 trials, lines indicate SE across the 5 trials). **(b)** Other females were not selective, responding similarly to the songs of many males (n = 1 bird, symbols as in panel (a)). **(c)** Across all birds that we tested, selectivity varied between the examples show in panels (a) and (b) (n = 12 birds; open circle indicates the bird in panel (a); open square indicates the bird shown in panel (b); different birds are indicated by different symbols; points indicate means of 5 trials, dotted line indicates level of chance).

**Fig 4:**
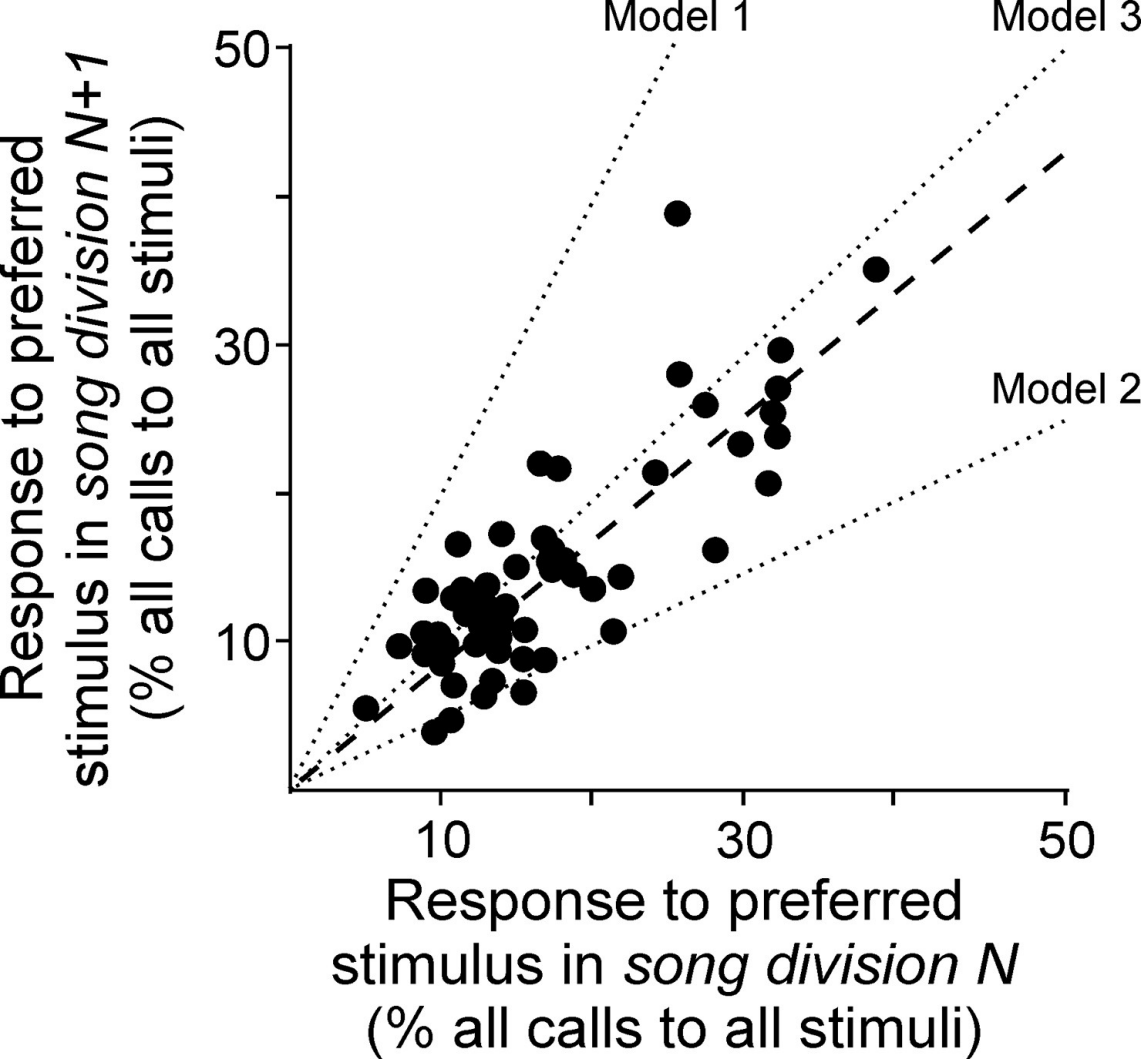
Behavioral indications of mate preference were consistent with female preference for some feature that is conserved throughout song performance. In our comparisons to investigate the relation between our dataset and predictions from each of three models (dotted lines, models detailed in the Methods), linear regression revealed a slope of 0.81 (dashed line, n = 12 birds), which was significantly different than zero (p < 0.001), and a y-intercept of 0.02 that was not significantly different than zero (p = 0.25). Each data point represents a test of song preference performed with an individual female (n = 5.25 ± 1.52 trials per female; for each natural song that was used to create experimental stimuli, each trial represents a division of that natural song at the level of halves, quarters, eighths, etc. as detailed in the Methods). The slope of our dataset (0.81) was significantly different than 2 (paired t-test, t = -7.69, df = 110, p < 0.001) and significantly different than ½ (paired t-test, t = 5.72, df = 110, p < 0.001) but not different than 1 (paired t-test, t = 0–1.10, df = 110, p = 0.27). Therefore, our data are most consistent with the Model 3, indicating that female song preference may be closely related to some feature that largely conserved throughout the song performance.

We quantified each female’s response to each song stimulus by counting the number of calls that the female performed in response to each stimulus. We used those data to quantify each bird’s response to the preferred stimulus at each level of song bisection by computing the response at each level of song dissection as a fraction of that bird’s total responses to all stimuli (e.g., Fig 3). We computed Pearson correlation coefficients to quantify the relation between response strengths at song dissection levels N and N+1 (e.g., Fig 4), and we used linear regression to compute the slope of those data. We used Student’s t-test to compare observed slope values versus slopes associated with each of the model predictions described above. We used paired t-tests to compare responses to songs with modified pitch or tempo versus the null hypothesis of no change in response. Finally, we used one-way ANOVAs to investigate whether song properties changed in our experimental manipulations of frequency or tempo. In all tests, significance was assessed at alpha = 0.05. All values are reported as means ± SE.

## Results

### Females express consistent preferences for individual males

Consistent with our previous study of female preference in this species [3], some females were very selective in their mate preference, as revealed by strong responses to the song of only 1 or 2 males and little or no response to songs of other males (e.g., Fig 3A). Other females were much less selective in their mate preference, as revealed by similar responses to the songs of many males (e.g., Fig 3B). For each female, there was a most-preferred song for which the female produced more calls than in response to any other stimulus (highest peaks in Fig 3A and 3B). Also consistent with our previous results [3], females varied in the identity of the song and singer that they found most attractive (among the 12 females tested, six different males were identified as a song that at least one female found most attractive). The 2 birds depicted in Fig 3A and 3B represent the most and the least selective birds that we sampled (n = 12 female birds, Fig 3C). Also consistent with our previous findings, female BFs were consistent in their preference across time and trials (detailed in Dunning et al. 2014). These findings provide the context in which we explored the degree to which changes in song properties were associated with changes in song preference.

### Female preference is related to features that are conserved throughout song duration

By plotting the strength of response at one level of song bisection (song division N in Fig 4) versus the strength of response at the next level of song bisection (song division N+1 in Fig 4), we could compare our data against predictions based on each of several possible relations between preference and song features (i.e., the models presented in Fig 4 and detailed in the Methods). Our data revealed a linear relation (linear regression, p < 0.001; slope = 0.83, n = 12 birds, Fig 4) in which the strength of response at each level of bisection was well correlated (Pearson R = 0.81, p < 0.01). If behavioral indicators of mate preference were closely related to a specific element or song stereotypy, we would expect a slope of 2 (Model 1 in Fig 4). Alternatively, if preference were closely related to song complexity or the diversity of different note types, then we would expect a slope of ½ (Model 2). Our data were not consistent with either of those possibilities playing a significant role in affecting female preference, as the slope of our data (0.81) was different than 2 (paired t-test, t = -7.69, df = 110, p < 0.001) and different than ½ (paired t-test, t = 5.72, df = 110, p < 0.001). These findings are consistent with previous observations that female BF song preference is not closely related to either note sequence or note sequence complexity [22, 24].

If mate preference were closely related to some feature that is conserved throughout the duration of the most-preferred song, then we would expect a slope of 1 (Model 3 in Fig 4). Our data were consistent with that idea, as the observed slope (0.81) was indistinguishable from a slope of 1 (Fig 4, paired t-test, t = -1.10, df = 110, p = 0.27). These data suggest that female BF mate preference is either closely related to some feature that is broadly conserved throughout song performances (Model 3), or female preference may be closely related to some combination of Models 1 and 2. We interpret these data to indicate that female BF preference may be related to some feature that is conserved throughout the song duration, as previous results indicate that female BF preference is not closely related to the parameters manipulated in Models 1 and 2 [22, 24].

### Female BF mate preference is predicted by note tempo but not by pitch

**Fig 5:**
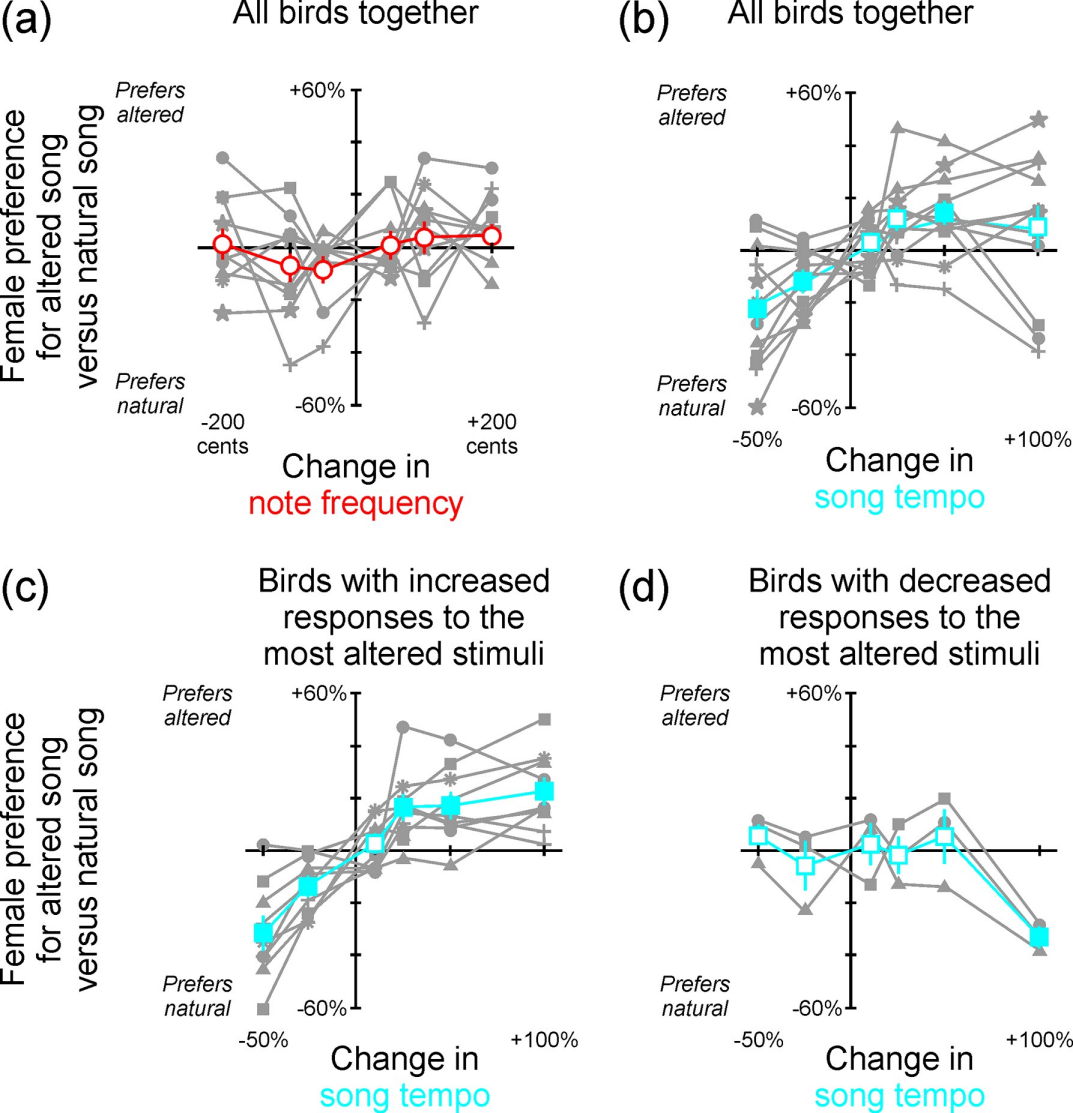
Female preference was related to changes in tempo but not note frequency. **(a)** Manipulating pitch had no systematic effect on female preference (n = 11 birds; individual birds are shown overlaid in gray using a different type of symbol for each bird; red line and large circles indicate the population response of all birds considered together; open symbols indicate cases of p ≥ 0.09 for paired t-tests comparing observed data versus model of no change; error bars are SE). **(b)** In contrast, manipulating tempo induced significant changes in preference (n = 12 birds, individual birds are shown overlaid in gray using a different type of symbol for each bird; blue line and large squares indicate the population response of all birds considered together; filled symbols indicate p < the Bonferroni-corrected 0.05 in tests as described in panel (a)). Altered preferences are evident at tempos that are present within the songs of male birds in the population where these females resided. When we divided birds according to whether their response to the maximally accelerated song was **(c)** greater than (n = 9 birds) or **(d)** less than (n = 3 birds) the response to the natural song, we observed that some birds preferred accelerated songs (panel (c)) but other birds preferred the natural song (panel (d)) (symbols as in panel (b)). No such differences were evident when data in panel (a) were subdivided according to their response to maximally increased or maximally decreased pitch.

To further investigate the degree to which female BF mate preference may be related to broadly conserved song features, we performed an additional manipulation to examine the degree to which female preference may be related to pitch or tempo of song notes. The pitch of each note type and the tempo in which notes are performed are broadly conserved throughout a song performance. Because individual notes were never divided or otherwise manipulated by our bisection technique, and because new stimuli were made with the same tempo as in the natural song (i.e., any silent intervals between subunits of song were equal to the mean internote interval of the natural song), pitch and tempo were conserved across bisections of the original song. Manipulation of pitch had little or no effect on the female’s song preference, evident as no cases in which manipulation induced a significant change in preference (Fig 5A, paired t-test comparing observed results against a model of no change in preference, p > 0.09 in all cases, n = 11 birds). These data reveal that note pitch is not a good predictor of female BF mate choice.

In contrast, tempo did have a significant impact on female song preference. Manipulations of tempo were associated with significant changes in the degree to which the females preferred the song (Fig 5B paired t-test comparing observed results against a model of no change in preference, p < Bonferroni-corrected 0.05 (0.01) indicated by filled symbols in Fig 5B, n = 12 birds). Females tended to prefer increased tempos (Fig 5B), but interestingly not all birds expressed the same preference. When we divided our sample according to each female’s preference for the most accelerated stimulus that we presented, we found that most birds expressed a preference for accelerated songs (Fig 5C, n = 9) but a few found highly accelerated songs to be less attractive than the natural song (Fig 5D, n = 3). The reasons for this individual-specific difference in song preference are not clear, as it was not the case that these three females preferred songs that were not preferred by any of the females in the other group of 9 birds. In addition, the tempos of the most-preferred song types for those 3 birds (8.34, 9.64 and 10.32 notes per second) were not restricted to either the fastest or the slowest tempos of songs in our set of stimuli, and those values fall within the natural variation in tempo that we have described previously for males in our population (range: 6.41 to 15.48 notes per sec; Table 1 in Dunning et al. 2014). When we used a similar approach to divide data collected in response to manipulation of pitch into different groups according to the nature of the response to maximally raised or maximally lower pitch, no trends were evident. Therefore, song preference in the females we studied was most closely related to tempo.

When we changed the tempo of the natural song, our manipulation left the species-typical song structure intact, but it also changed the frequency modulation and duration of individual notes. To disambiguate the influence of tempo (notes/sec) from the influence of those changes in note properties, we presented a set of stimuli in which we took the notes of the 50% increased tempo stimulus (one for which females expressed a significant increase in preference, Fig 5B) and increased the duration of silence between each note so that the tempo of the new stimulus was the same as in the natural song. When we presented those stimuli, females expressed an increased preference for songs containing alteration of both tempo and note properties (S1 Fig, paired t-test comparing responses to natural versus altered versions of the female’s most-preferred song type, t = 3.23, df = 11, p = 0.008) but not for songs containing alterations of note properties but not tempo (tempo was the same as in the natural song; S1 Fig; paired t-test comparing responses to natural versus altered versions of the female’s most-preferred song type, t = -0.71, df = 11, p = 0.49). These data further support the idea that tempo plays an important role in shaping female song preference.

As a final examination of the role of tempo in mate preference, we asked whether preference was more closely related to absolute tempo or relative change from the natural tempo. We addressed that by computing the strength of correlation between female preference and absolute tempo (R^2^ = 0.17, p < 0.001) and between female preference and the relative change in tempo (Pearson R^2^ = 0.25, p < 0.001). Although this difference is subtle, these data reveal that preference is more closely associated with relative changes in tempo. Therefore, it is not simply the case that the fastest song that the female ever hears is the most attractive song. Instead, it appears that female mate preference is more closely related to the degree to which tempo is increased in the context of other unchanged song parameters.

## Discussion

Female BFs use song to identify and select a male as their mate [3]. Here we identified a property of male song that informs a female’s evaluation of song quality. Our approach enabled us to assess the possible contribution of a wide variety of song properties, however the results indicated that female BF preference is influenced by a specific parameter of song performance. Female preference was strongly affected by the degree to which we changed tempo from its natural value. Our data do not preclude the possibility that other factors, such as song duration or other traits not tested here, may also play a role in affecting female BF preference. In fact, our results leave open the possibility that some combination of features in Model 1 and Model 2 may interact such that female preference is not related to either of those features individually but they nonetheless play a role that we did not detect due to the sensitivity or design of the experimental methods. Thus, our data should not be taken as evidence of no role for those features. Instead, our data reveal that no single trait tested in those models is closely related to female song preference. However, our data do reveal a feature of male song that does play a significant role in affecting female mate preference. Female BF mate preference can be altered by experimentally manipulating the tempo of male song. That relation between preference and tempo was evident both within and outside of the range of naturally occurring tempos (natural ranges of a wide range of song properties among males in our population are detailed in Dunning et al. 2014), and most females preferred accelerated songs.

In addition to highlighting tempo as a salient feature of male song, this result also clarifies our interpretation of the data in the models presented in Fig 4. Our data were not consistent with either Model 1 (slope = 2, which would have indicated an important role for a specific song element or the stereotypy of note content or sequence) or Model 2 (slope = ½, which would have indicated an important role for the number of different note types or the complexity of note content or sequence), but our data were consistent with Model 3 (slope = 1, which suggests an important role for feature(s) that are broadly conserved throughout song performance). We interpreted those results as contradictory of Models 1 and 2 and supportive of Model 3. An alternative explanation, however, is that data with a slope of approximately 1 could reflect some combination of influences from Models 1 and 2 rather than the influence of some property that is conserved throughout the song. If it were the case that preference is closely related to a combination of Models 1 and 2 but not related to Model 3 (i.e., mate preference is strongly related to that combination but not to any other song feature), then we should not have observed a change in preference in response to changes in tempo. In experimental tests of the role of note sequence complexity in affecting female BF mate preference, others have also detected little or no relationship between preference and that aspect of male song [22, 24]. Our results from bisecting songs and assembling new stimuli from each half are consistent with those previous observations. The fact that we observed significant changes in female preferences when we manipulated tempo supports our interpretation that preference is related to a property that is conserved throughout the song.

### Considering salient features of BF song in light of salient features in other species

Female mate preference has been linked to a variety of song features. In some species, females are attracted to behavioral diversity, such as the size of a male’s repertoire of different songs or note types [11, 13]. In other species, females appear to prefer songs that are more stereotyped in their content [21]. In other cases, females are attracted to specific song elements, such as the “sexy syllables” that are attractive to female canaries [14, 15], accelerated tempos that are attractive to female Lincoln’s sparrows [17], and the combination of frequency bandwidth and the speed at which song elements are performed that is attractive to female swamp sparrows [18]. Our data indicate that in the BFs that we tested, female mate preference is not closely related to song parameters such as the diversity of note types, the complexity of note sequence, or the stereotypy of song performance. Instead, our data implicate relative changes in tempo as playing an important role in female mate choice.

Although these findings may appear to implicate a single parameter in song evaluation, a closer examination of our data and results from other species suggests that female BFs are evaluating song in much the same way as females of other species. For example, the “sexy syllables” in canary songs are especially salient in shaping female preference. Removing those syllables from a song that a female prefers results in her degree of preference being strongly reduced or eliminated [14, 15]. If a song stimulus is created using only sexy syllables, then females express a strong preference for that synthetic song [14, 15]. Therefore, sexy syllables are both necessary and sufficient to make a song attractive to a female canary. In those especially salient elements of canary song, each syllable consists of a two-note structure in which the notes span a large frequency bandwidth and are performed in rapid succession. Each of those properties is important, as syllables that females find attractive are characterized by frequency modulation between ~ 800 and ~ 4000 Hz and internote intervals less than ~ 23 to 25 ms [14, 15]. Therefore, the attractive quality of the sexy syllable requires that a large bandwidth is performed rapidly.

The preference of female canaries for a combination of spectral and temporal elements of male song is strikingly similar to the preferences expressed by female swamp sparrows. Female swamp sparrows prefer songs that lie at a performance limit of frequency bandwidth and trill rate [18, 26]. In both swamp sparrows and canaries, notes performed in rapid succession are attractive, but only if those notes contain a wide bandwidth, and it is thought that this combination of rapid performance and wide bandwidth is challenging to produce. In Lincoln’s sparrows (*Melospiza lincolnii*), females also prefer songs in which trill rate has been experimentally increased, and it is thought that such cases of increased tempo may also be challenging for males to produce [17]. In the female BF’s described here, mate choice is most closely related to the degree to which tempo has increased while not altering the bandwidth of individual notes. Control tests eliminated the possibility that mate preference is strongly influenced by a change in frequency modulation or note duration. Therefore, it appears that BFs share similarities with wild songbird species in that many traits may influence female mate preference, but tempo plays an especially important role.

### Considering possible benefits associated with female preference for increased tempo

Rapid performance of notes that contain wide frequency bandwidth serves as an honest signal of male quality because it is difficult to fake and costly to produce [27]. Sweeping through song frequencies requires precise control of the muscles that control the vocal organ and beak [28], and the ability to sweep through large bandwidths rapidly indicates that a male bird has developed and maintained high-quality versions of that musculature and the associated neural control centers. The nutritional stress hypothesis, originally proposed by Nowicki and colleagues using swamp sparrows and later extended to zebra finches by Catchpole and colleagues, posits that it is costly for males to build and maintain those song control structures [29, 30]. If a male is deprived of adequate nutrition during development, then the neural structures underlying song performance will not develop as well [31], the quality of that male’s song will be negatively impacted, and it will be less attractive to females [30, 32, 33]. In contrast, well-fed juvenile males develop songs that females find more attractive, and some authors have speculated that song quality may also be correlated with better performance in other measures of cognition. Several studies have reported correlations between male nutritional status, physiological status, brain volume and song quality [33–36]. Therefore, song is a behavioral ornament that indicates sex and species identity, is closely linked to the development and maintenance of complex neural circuitry, and may also provide the listener with information about the male’s quality. By selecting for a male with superior performance in both tempo and bandwidth, a female may be selecting for high-quality males that were provided adequate resources during development and that have been successful in building and maintaining the neural components underlying song performance and other beneficial behaviors such as foraging.

Song performance may also be signaling additional aspects of the male’s current condition. For example, the degree to which male swamp sparrows can perform physically challenging songs is an indicator of the male’s age and size [37]. In male BFs, age is related to song tempo, with older males singing slower songs [38]. Older males do not show deterioration of the associated muscles, leading to speculation that slower song emerges as a result of deterioration of the underlying neural systems [38]. BF males also become less variable in their note sequence as they age [39], and songs have apparently become more complex as white-rumped munias have been domesticated into BFs [40]. Our data did not reveal a significant role for sequence variability in affecting female evaluation of song quality, but it could provide females an additional means of assessing the age of their suitor.

We were initially surprised to detect a relation between female mate choice and spectrotemporal performance in our population. BFs and domestic canaries are captive species and swamp sparrows and Lincoln’s sparrows are wild, yet our data suggest a similar basis of song evaluation in all four species. Our data reveal a broadly relevant pattern of song evaluation in a species that is especially useful for neurobiological studies in the laboratory [e.g., 41, 42], opening the door to additional investigations of how preferences develop, how they are encoded in auditory processing, and how that information is used to direct selective mate choice behaviors. Curiously, not all of the females that we sampled expressed the same preference for accelerated tempo. Three birds found accelerated songs less attractive. Previous studies have revealed differences among BF females in what they find attractive, such that the preference of an individual is consistent across time and tests, but individual females vary and what they find attractive [3]. It is not clear from the present data why these three females did not follow the population trend. One possibility is that aspects of their social experience or their adult status could have contributed to these atypical preferences [43–45], but additional studies are needed to determine the source of these differences and whether those individual-specific preferences may be adaptive or maladaptive.

These results demonstrating female preference for male songs that are challenging to perform are consistent with emerging theories in behavioral ecology that also suggest that female mate choice is heavily influenced by male motor performances. Specifically, vigor is an honest measure of a male’s ability to sustain repeated performance of difficult actions [27]. Vigorous or extreme displays require large amounts of metabolic output and likely provide information to female perceivers because a male’s performance is constrained by limitations in the power capacities of the associated muscles [46]. Byers [27] describes vigor as the summed expression of an organism’s functional genome, capturing aspects of growth and development, limb proportions, immune function, parasite load, and neural systems underlying motivation and performance. Studies in pronghorn [47], hummingbirds [48], wolf spiders [49], fireflies [50], golden-collared manakins [51], and woodpeckers [52] highlight male vigor and sustained, repetitive motor performances as a common denominator in mating strategies across animal taxa. Results of the present study provide additional empirical support for vigorous male performance of courtship signals playing a key role in shaping the preferences of female receivers.

## Supporting information

S1 Fig Female preference was related to changes in tempo. When we compared females’ responses to their most-preferred song type versus experimentally altered versions of that song, females expressed significant preference for songs in which tempo and frequency modulation were both altered (left, n = 12 birds, paired t-test, t = 3,23, df = 11, p = 0.008, indicated by asterisk) but not for songs in which frequency modulation was altered but tempo was the same as in the natural song (t = -0.71, df = 11, p = 0.49). These data support the idea that tempo plays an important role in affecting female evaluation of song quality.
(TIF)

## References

[1] Catchpole CK, Slater PJB. *Birdsong*: *Biological Themes and Variations*. 2nd. ed Cambridge: Cambridge University Press; 2008.

[2] Searcy W, Brenowitz E. Sexual differences in species recognition of avian song. Nature. 1988;332:152–4.

[3] Dunning J, Pant S, Bass A, Coburn Z, Prather J. Mate choice in adult female Bengalese finches: females express consistent preferences for individual males and prefer female-directed song performances. Plos One. 2014;9(2):e89438 10.1371/journal.pone.0089438 24558501 PMC3928452

[4] Searcy W. Measureing responses of female birds to male song In: McGregor P, editor. Playback and Studies of Animal Communication. New York: Plenum Press; 1992 p. 175–89.

[5] Gentner TQ, Hulse SH. Female European starling preference and choice for variation in conspecific male song. Anim Behav. 2000;59(2):443–58. Epub 2000/02/17. 10.1006/anbe.1999.1313 . 10675267

[6] Clayton N, Prove E. Song Discrimination in Female Zebra Finches and Bengalese Finches. Anim Behav. 1989;38:352–4. ISI:A1989AJ88700017.

[7] Eens M, Pinxten R, Verheyen RF. Male Song as a Cue for Mate Choice in the European Starling. Behaviour. 1991;116:210–38. 10.1163/156853991x00049 ISI:A1991FC16100004.

[8] Kempenaers B, Verheyren GR, Dhondt AA. Extrapair paternity in the blue tit (Parus caeruleus): female choice, male characteristics, and offspring quality. Behav Ecol. 1997;8(5):481–92. 10.1093/beheco/8.5.481 ISI:A1997XX33800003.

[9] Wasserman FE, Cigliano JA. Song Output and Stimulation of the Female in White-Throated Sparrows. Behav Ecol Sociobiol. 1991;29(1):55–9. 10.1007/Bf00164295 ISI:A1991GC05400008.

[10] Hasselquist D, Bensch S, vonSchantz T. Correlation between male song repertoire, extra-pair paternity and offspring survival in the great reed warbler. Nature. 1996;381(6579):229–32. 10.1038/381229a0 ISI:A1996UL24900052.

[11] Catchpole CK. Song Repertoires and Reproductive Success in the Great Reed Warbler Acrocephalus-Arundinaceus. Behav Ecol Sociobiol. 1986;19(6):439–45. 10.1007/Bf00300547 ISI:A1986F110500008.

[12] Marler P, Slabbekoorn H. Nature's music: the science of birdsong. San Diego, CA: Elsevier Academic Press; 2004.

[13] Reid JM, Arcese P, Cassidy ALEV, Hiebert SM, Smith JNM, Stoddard PK, et al Song repertoire size predicts initial mating success in male song sparrows, Melospiza melodia. Anim Behav. 2004;68:1055–63. 10.1016/j.anbehav.2004.07.003 ISI:000225064400009.

[14] Vallet E, Kreutzer M. Female Canaries Are Sexually Responsive to Special Song Phrases. Anim Behav. 1995;49(6):1603–10. ISI:A1995RE98700020.

[15] Vallet E, Beme I, Kreutzer M. Two-note syllables in canary songs elicit high levels of sexual display. Anim Behav. 1998;55:291–7. ISI:000072105500003. 10.1006/anbe.1997.0631 9480696

[16] Rehsteiner U, Geisser H, Reyer HU. Singing and mating success in water pipits: one specific song element makes all the difference. Anim Behav. 1998;55:1471–81. 10.1006/anbe.1998.0733 ISI:000074936600007. 9641992

[17] Caro SP, Sewall KB, Salvante KG, Sockman KW. Female Lincoln's sparrows modulate their behavior in response to variation in male song quality. Behav Ecol. 2010;21(3):562–9. Epub 2010/05/01. 10.1093/beheco/arq022 22476505 PMC2854529

[18] Ballentine B, Hyman J, Nowicki S. Vocal performance influences female response to male bird song: an experimental test. Behav Ecol. 2004;15(1):163–8. 10.1093/beheco/arg090 ISI:000189345400020.

[19] Nowicki S, Searcy WA. Song function and the evolution of female preferences—Why birds sing, why brains matter. Ann Ny Acad Sci. 10162004 p. 704–23. 10.1196/annals.1298.012 15313801

[20] Sossinka R, Bohner J. Song Types in the Zebra Finch Poephila-Guttata-Castanotis. Zeitschrift Fur Tierpsychologie-Journal of Comparative Ethology. 1980;53(2):123–32. ISI:A1980KN94100002.

[21] Woolley SC, Doupe AJ. Social context-induced song variation affects female behavior and gene expression. PLoS Biol. 2008;6(3):e62 Epub 2008/03/21. 07-PLBI-RA-1260 [pii ] 10.1371/journal.pbio.0060062 18351801 PMC2267820

[22] Morisaka T, Katahira K, Okanoya K. Variability in preference for conspecific songs with syntactical complexity in female Bengalese Finches: towards an understanding of song evolution. Ornithol Sci. 2008;7:75–84.

[23] Woodgate JL, Mariette MM, Bennett ATD, Griffith SC, Buchanan KL. Male song structure predicts reproductive success in a wild zebra finch population. Anim Behav. 2012;83(3):773–81. 10.1016/j.anbehav.2011.12.027 ISI:000300618100023.

[24] Kato Y, Hasegawa T, Okanoya K. Song preference of female Bengalese finches as measured by operant conditioning. J Ethol. 2010;28(3):447–53. 10.1007/s10164-010-0203-7 ISI:000280088700005.

[25] Mooney R, Prather JF, Roberts T. Neurophysiology of Birdsong Learning In: Eichenbaum H, editor. Learning and Memory: A Comprehensive Reference. Vol. 3 Memory Systems. Oxford: Elsevier; 2008 p. 441–74.

[26] Podos J. Motor constraints on vocal development in a songbird. Anim Behav. 1996;51:1061–70. ISI:A1996UQ64900009.

[27] Byers J, Hebets E, Podos J. Female mate choice based upon male motor performance. Anim Behav. 2010;79(4):771–8. 10.1016/j.anbehav.2010.01.009 ISI:000275801500001.

[28] Podos J, Sherer JK, Peters S, Nowicki S. Ontogeny of Vocal-Tract Movements during Song Production in Song Sparrows. Anim Behav. 1995;50:1287–96. ISI:A1995TE32500015.

[29] Nowicki S, Searcy WA, Peters S. Brain development, song learning and mate choice in birds: a review and experimental test of the 'nutritional stress hypothesis'. J Comp Phys A. 2002;188:1003–14. 10.1007/s00359-002-0361-3 12471497

[30] Spencer KA, Buchanan KL, Goldsmith AR, Catchpole CK. Song as an honest signal of developmental stress in the zebra finch (Taeniopygia guttata). Horm Behav. 2003;44(2):132–9. 10.1016/s0018-506x(03)00124-7 ISI:000185305800006. 13129485

[31] Honarmand M, Thompson CK, Schatton A, Kipper S, Scharff C. Early developmental stress negatively affects neuronal recruitment to avian song system nucleus HVC. Dev Neurobiol. 2016;76(1):107–18. Epub 2015/05/20. 10.1002/dneu.22302 . 25980802

[32] Buchanan KL, Spencer KA, Goldsmith AR, Catchpole CK. Song as an honest signal of past developmental stress in the European starling (Sturnus vulgaris). P Roy Soc Lond B Bio. 2003;270(1520):1149–56. 10.1098/rspb.2003.2330 ISI:000183400900007. 12816653 PMC1691349

[33] Nowicki S, Peters S, Podos J. Song learning, early nutrition and sexual selection in songbirds. Am Zool. 1998;38(1):179–90. ISI:000073037900014.

[34] Pravosudov VV, Lavenex P, Omanska A. Nutritional deficits during early development affect hippocampal structure and spatial memory later in life. Behav Neurosci. 2005;119(5):1368–74. 10.1037/0735-7044.119.5.1368 ISI:000233210700019. 16300442

[35] MacDonald IF, Kempster B, Zanette L, MacDougall-Shackleton SA. Early nutritional stress impairs development of a song-control brain region in both male and female juvenile song sparrows (Melospiza melodia) at the onset of song learning. P Roy Soc B-Biol Sci. 2006;273(1600):2559–64. 10.1098/rspb.2006.3547 ISI:000240729900021. 16959649 PMC1634898

[36] Wainwright PE, Colombo J. Nutrition and the development of cognitive functions: interpretation of behavioral studies in animals and human infants. Am J Clin Nutr. 2006;84(5):961–70. ISI:000241937700003. 10.1093/ajcn/84.5.961 17093144

[37] Ballentine B. The ability to perform physically challenging songs predicts age and size in male swamp sparrows, Melospiza georgiana. Anim Behav. 2009;77(4):973–8. 10.1016/j.anbehav.2008.12.027 ISI:000264325300025.

[38] Cooper BG, Mendez JM, Saar S, Whetstone AG, Meyers R, Goller F. Age-related changes in the Bengalese finch song motor program. Neurobiology of Aging. 2012;33(3):564–8. 10.1016/j.neurobiolaging.2010.04.014 ISI:000299786000013. 20570409 PMC2957555

[39] James LS, Sakata JT. Vocal motor changes beyond the sensitive period for song plasticity. J Neurophysiol. 2014;112(9):2040–52. Epub 2014/07/25. jn.00217.2014 [pii] 10.1152/jn.00217.2014 25057147 PMC4274927

[40] Katahira K, Suzuki K, Kagawa H, Okanoya K. A simple explanation for the evolution of complex song syntax in Bengalese finches. Biol Letters. 2013;9(6). Artn 20130842 ISI:000330290400044. 10.1098/rsbl.2013.0842 PMC3871373 24284561

[41] Dunning JL, Maze SE, Atwood EJ, Prather JF. Caudal mesopallial neurons in female songbirds bridge sensory and motor brain regions. J Comp Neurol. 2018;526(10):1703–11. Epub 2018/04/01. 10.1002/cne.24440 . 29603218

[42] Elie J, Hoffmann S, Dunning J, Coleman M, Fortune E, Prather J. From perception to action: the role of auditory input in shaping vocal communication and social behaviors. Brain Behav Evol. in press. 10.1159/000504380 31805560

[43] Holveck MJ, Riebel K. Female zebra finches learn to prefer more than one song and from more than one tutor. Anim Behav. 2014;88:125–35. 10.1016/j.anbehav.2013.11.023 ISI:000331134500016.

[44] Nagle L, Kreutzer ML. Adult female domesticated canaries can modify their song preferences. Can J Zool. 1997;75(8):1346–50. ISI:A1997XP66500019.

[45] Burley NT, Foster VS. Variation in female choice of mates: condition influences selectivity. Anim Behav. 2006;72:713–9. 10.1016/j.anbehav.2006.01.017 ISI:000240730300025.

[46] Clark CJ. The role of power versus energy in courtship: what is the “energetic cost” of a courtship display? Anim Behav. 2012;84:269–77.

[47] Byers JA, Moodie JD, Hall N. Pronghorn females choose vigorous mates. Anim Behav. 1994;47:33–43.

[48] Stiles FG. Aggressive and courtship displays of the male Anna’s hummingbird. Condor. 1982;84:208–25.

[49] Hebets EA, Uetz GW. Female responses to isolated signals from multimodal male courtship displays in the wolf spider genus Schizocosa (Araneae: Lycosidae). Anim Behav. 1999;57:865–72. 10.1006/anbe.1998.1048 10202094

[50] Lewis SM, Cratsley CK. Flash signal evolution, mate choice, and predation in fireflies. Annual Review of Entomology. 2008;53:293–321. 10.1146/annurev.ento.53.103106.093346 17877452

[51] Barske J, Schlinger BA, Wikelski M, Fusani L. Female choice for male motor skills. Proceedings of the Royal Society of London B: Biological Sciences. 2011;278:3523–8. 10.1098/rspb.2011.0382 PMC3189371 21508030

[52] Schuppe ER, Fuxjager MJ. High‐speed displays encoding motor skill trigger elevated territorial aggression in downy woodpeckers. Functional Ecology. 2017;32(2):450–60.

