# Supplementary figures and images for: Female finches prefer courtship signals indicating male vigor and neuromuscular ability
Source: PLoS One. 2020 Jan 10;15(1):e0226580. doi: 10.1371/journal.pone.0226580 (PMC6953821; doi:10.1371/journal.pone.0226580)

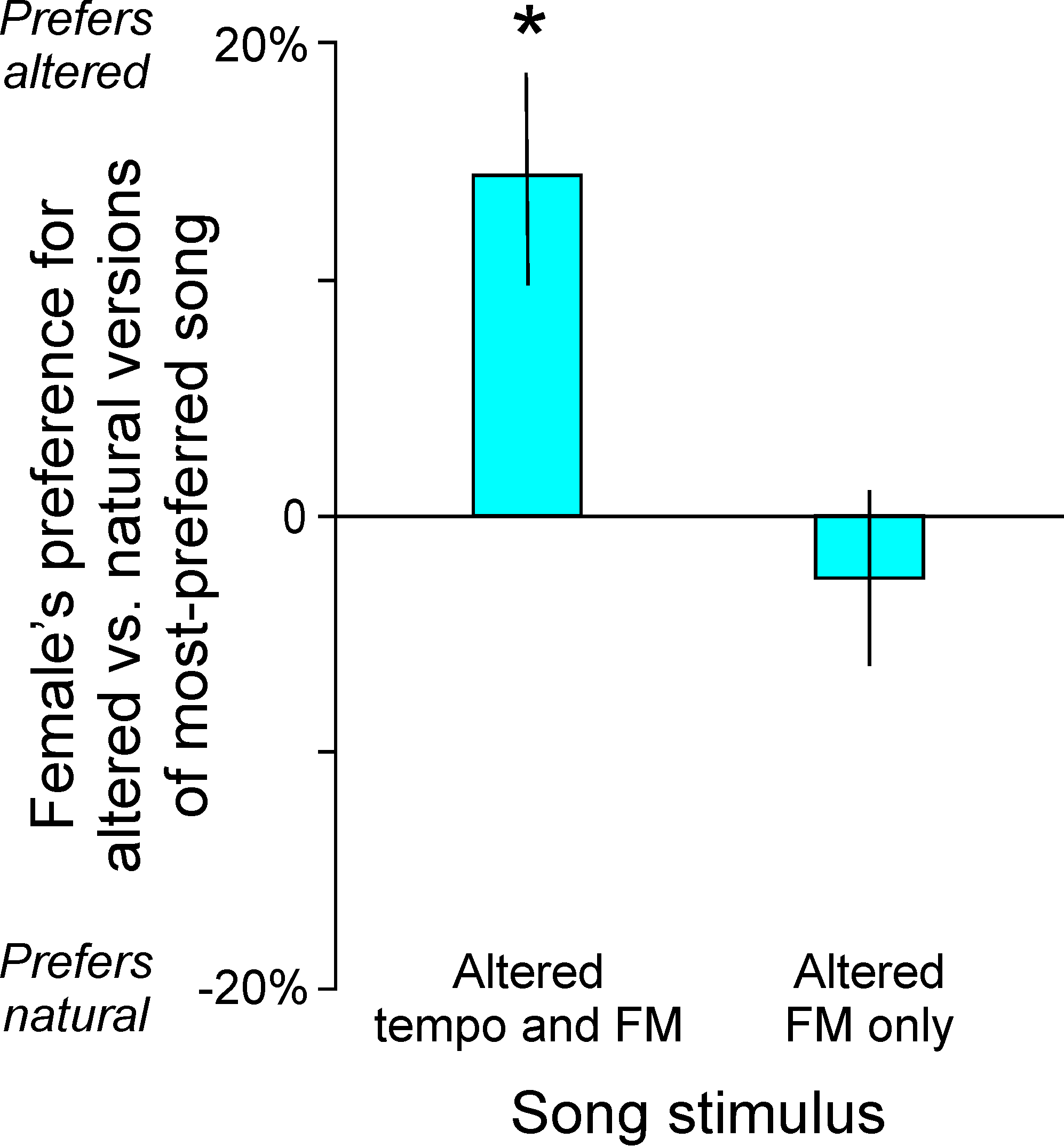

Supplement: S1 Fig — When we compared females’ responses to their most-preferred song type versus experimentally altered versions of that song, females expressed significant preference for songs in which tempo and frequency modulation were both altered (left, n = 12 birds, paired t-test, t = 3,23, df = 11, p = 0.008, indicated by asterisk) but not for songs in which frequency modulation was altered but tempo was the same as in the natural song (t = -0.71, df = 11, p = 0.49). These data support the idea that tempo plays an important role in affecting female evaluation of song quality. (TIF) [file pone.0226580.s001.tif]
